# Genotype-phenotype correlations of amyotrophic lateral sclerosis

**DOI:** 10.1186/s40035-016-0050-8

**Published:** 2016-02-03

**Authors:** Hong-Fu Li, Zhi-Ying Wu

**Affiliations:** Department of Neurology and Research Center of Neurology in Second Affiliated Hospital, and the Collaborative Innovation Center for Brain Science, Zhejiang University School of Medicine, 88 Jiefang Rd, Hangzhou, 310009 China

**Keywords:** Amyotrophic lateral sclerosis, Diagnosis of ALS, Causative genes, Genetic explanations, Genotype-phenotype correlations

## Abstract

Amyotrophic lateral sclerosis (ALS) is a devastating neurodegenerative disease characterized by progressive neuronal loss and degeneration of upper motor neuron (UMN) and lower motor neuron (LMN). The clinical presentations of ALS are heterogeneous and there is no single test or procedure to establish the diagnosis of ALS. Most cases are diagnosed based on symptoms, physical signs, progression, EMG, and tests to exclude the overlapping conditions. Familial ALS represents about 5 ~ 10 % of ALS cases, whereas the vast majority of patients are sporadic. To date, more than 20 causative genes have been identified in hereditary ALS. Detecting the pathogenic mutations or risk variants for each ALS individual is challenging. However, ALS patients carrying some specific mutations or variant may exhibit subtly distinct clinical features. Unraveling the respective genotype-phenotype correlation has important implications for the genetic explanations. In this review, we will delineate the clinical features of ALS, outline the major ALS-related genes, and summarize the possible genotype-phenotype correlations of ALS.

## Background

Amyotrophic lateral sclerosis (ALS) is a devastating and inexorable neurodegenerative disease characterized by progressive neuronal loss and degeneration of upper motor neuron (UMN) and lower motor neuron (LMN) [1]. It is the most widespread type of motor neuron disease and has become the third most common neurodegenerative disease in the world [2]. Patients affected with ALS typically suffer from progressive muscle weakness and atrophy and usually die from respiratory failure 2 to 3 years after the onset [3]. ALS is a relentless and incurable disease. There has been no effective therapeutic approach to halt the progression so far. ALS is heterogeneous in its presentation, course, and progression. No single test for diagnosing ALS exists; most cases are diagnosed based on symptoms, physical signs, progression, EMG, and tests to exclude the overlapping conditions.

The etiology of ALS is not fully understood. Appropriate 5 ~ 10 % of ALS is familial (FALS) with a Mendelian pattern of inheritance, suggesting that genetic factors play important roles in the pathogenesis of ALS [4]. To date, more than 20 causative genes have been identified in hereditary ALS. In addition, about 30 potential causative or disease-modifying genes have also been identified. Detecting the pathogenic mutations or risk variants for each ALS individual is therefore challenging. Clinical features do not reliably separate the hereditary ALS from sporadic ALS (SALS) due to phenotypic overlap. However, ALS patients carrying some specific mutations may exhibit subtly distinct clinical features. Unraveling the genotype-phenotype correlations has important implications for the genetic explanations. In this review, we will delineate the clinical features of ALS, outline the major ALS-related genes, and review the possible genotype-phenotype correlations. We hope this review will be in favor of improving the accuracy of genetic screenings.

## Clinical features of ALS

ALS usually commences in later life, with a mean age at onset (AAO) of 65 year. The onset for FALS tends to be earlier than that of SALS. A small proportion of patients may developed juvenile ALS (JALS), in which onset occurs in the first two decades [5]. Symptoms usually begin in the limbs (termed limb onset), although approximate 25 % of ALS patients have bulbar onset. Associated with poorer prognosis, bulbar onset is more common in elderly patients and women [6]. Upper limb weakness and atrophy at onset are most common, subsequently spreading to involve bulbar, trunk, or respiratory muscle [7]. After initial presentation of symptoms, the disease progresses to include both UMN and LMN symptoms and signs. The UMN disturbance involving limbs leads to spasticity, weakness, and brisk reflexes. The LMN features of limbs comprise muscle atrophy, weakness, fasciculation, and decreased reflexes. As limb function deteriorates, patients gradually lose their ability to walk. Bulbar UMN dysfunction includes spastic dysarthria and brisk jerk of gag and jaw, while bulbar LMN dysfunction includes tongue wasting, weakness, and fasciculation. In the later stage of ALS, most cases develop dysphagia, which are associated with weight loss and malnutrition.

Sensory loss is usually absent, but cognitive impairment is common in ALS cases. A significant proportion of ALS cases develop cognitive dysfunction, and in a minority, overt dementia [7]. With appropriate cognitive assessment, 20 ~ 50 % of patients with ALS fulfill the consensus criteria for probable or definite frontal temporal dementia (FTD) [8]. In addition, about 30 % of FTD patients manifest signs of motor system dysfunction [9]. The occurrence of ALS, FTD, or ALS with FTD in intra-familial members discloses a considerable overlap between ALS and FTD.

## Causative genes

Since the identification of first causative gene in 1993, a growing number of ALS-causing genes associated with Mendelian inheritance have been identified. Although FALS represents only 5 ~ 10 % of ALS cases, investigations of the causative genes have greatly increased our understanding of the etiology of ALS. FALS is generally inherited in an autosomal dominant pattern and rarely inherited as an autosomal recessive or X-linked trait [10]. Adult onset autosomal dominant inheritance is more common than juvenile onset which is usually caused by recessive transmission.

To date, at least 21 chromosomal regions containing 19 identified genes have been linked to ALS, termed as ALS 1–21 respectively (Table 1). In 2011, the *chromosome 9 open reading frame 72* (*C9ORF72*) repeat expansions were identified in a significant proportion of ALS and FTD patients, becoming the most common genetic cause of ALS in the Caucasian population. There has been strong evidence to support the pathogenic role for mutations in *Cu/Zn superoxide dismutase 1* (*SOD1*), *Fused in sarcoma* (*FUS*), *TAR DNA-binding protein* (*TARDBP*), and *C9ORF72*. However, mutations in other genes are not common or even rarely seen in ALS cases. In addition to the aforementioned ALS genes, a constellation of other genes have also been implicated in ALS [11]. But mutations in these genes are only identified in a small fraction of ALS patients, indicating that these mutated genes have little contribution to the development of ALS.

**Table 1:** Different subtypes of familial ALS and their genotype–phenotype correlations

ALS type	Gene	Inheritance	ALS features	FTD	Other features/disorders
(chromosome)					
ALS 1	*SOD1*	AD; AR;	AAO: adult > juvenile; Onset: LL > UL > bulbar;	Rare	PMA, PBP, BFA, cerebellar ataxia, autonomic dysfunction
(21q22.1)		De novo	Progression: rapid > slow; UMN + LMN > LMN dominant		
ALS 2	*ALSIN*	AR	AAO: juvenile; Onset: LL, UL;	None	PLS, IAHSP
(2q33.2)			Progression: slow; UMN dominant > UMN + LMN		
ALS 3	UN	AD	N/A	N/A	N/A
(18q21)					
ALS 4	*SETX*	AD	AAO: juvenile > adult; Onset: LL > UL;	None	AOA2, cerebellar ataxia, motor neuropathy
(9q34)			Progression: slow; UMN + LMN > LMN dominant		
ALS 5	*SPG11*	AR	AAO: juvenile > adult; Onset: bulbar, limb;	Rare	HSP, autonomic dysfunction, mental retardation
(15q21.1)			Progression: slow; UMN dominant > UMN + LMN		
ALS 6	*FUS*	AD; AR;	AAO: adult > juvenile; Onset: UL, bulbar > LL;	Rare	PMA, Parkinsonism, essential tremor, mental retardation
(16q11.2)		De novo	Progression: rapid > slow; UMN + LMN > LMN dominant		
ALS 7	UN	AD	N/A	N/A	N/A
(20p13)					
ALS 8	*VAPB*	AD	AAO: adult > juvenile; Onset: limb;	None	SMA, motor neuropathy, autonomic dysfunction
(20q13.3)			Progression: slow; LMN dominant		
ALS 9	*ANG*	AD	AAO: adult > juvenile; Onset: limb, bulbar;	Yes	PBP, PD
(14q11.2)					
			Progression: N/A; UMN + LMN		
ALS 10	*TARDBP*	AD; AR	AAO: adult; Onset: limb, bulbar;	Yes	PSP, FTD with Parkinsonism, PD, chorea
(1p36.22)			Progression: variable; UMN + LMN		
ALS 11	*FIG4*	AD	AAO: adult; Onset: bulbar > limb;	None	CMT4J, HSP, PLS, Yunis–Varon syndrome, epilepsy with polymicrogyria
(6q21)			Progression: variable; UMN + LMN > UMN dominant		
ALS 12	*OPTN*	AD; AR	AAO: adult; Onset: bulbar, limb;	Yes	POAG, Parkinsonism, aphasia
(10p13)			Progression: slow; UMN + LMN		
ALS 13	*ATXN2*	AD	AAO: adult > juvenile; Onset: UL, LL;	None	SCA2, Parkinsonism
(12q24)			Progression: variable; UMN + LMN		
ALS 14	*VCP*	AD	AAO: adult > juvenile; Onset: limb > bulbar;	Yes	IBMPFD
(9p13)			Progression: variable; UMN + LMN		
ALS 15	*UBQLN2*	XD	AAO: adult > juvenile; Onset: limb, bulbar;	Yes	PLS
(Xp11.21)			Progression: variable; UMN + LMN > UMN dominant		
ALS 16	*SIGMAR1*	AD	AAO: juvenile; Onset: LL > UL;	Rare	motor neuropathy
(9p13.3)			Progression: N/A; UMN + LMN		
ALS 17	*CHMP2B*	AD	AAO: adult; Onset: bulbar, limb;	Yes	PMA; Parkinsonism
(3p12.1)			Progression: N/A; UMN + LMN > LMN dominant		
ALS 18	*PFN1*	AD	AAO: adult; Onset: limb;	None	N/A
(17p13.2)			Progression: N/A; UMN + LMN		
ALS 19	*ERBB4*	AD	AAO: adult; Onset: UL, bulbar;	None	N/A
(2q33.3-q34)			Progression: slow; UMN + LMN		
ALS 20	*hnRNPA1*	AD	AAO: adult; Onset: N/A;	Yes	multisystem proteinopathy
(12q13.1)			Progression: N/A; UMN + LMN > LMN dominant		
ALS 21	*MATR3*	AD	AAO: adult; Onset: bulbar, limb;	Yes	distal myopathy
(5q31.3)			Progression: slow; UMN + LMN > LMN dominant		
ALS-FTD	*C9ORF72*	AD	AAO: adult; Onset: bulbar, limb;	Yes	Parkinsonism, cerebellar ataxia
(9p21.2)			Progression: rapid > slow; UMN + LMN		

*Abbreviations: AAO* age at onset, *AD* autosomal dominant, *ALS* amyotrophic lateral sclerosis, *AOA2* ataxia and oculomotor apraxia type 2, *AR* autosomal recessive, *BFA* benign focal amyotrophy, *CMT4J* Charcot-Marie-Tooth disease, *FTD* frontotemporal dementia, *HSP* hereditary spastic paraplegia, *IAHSP* infantile-onset ascending hereditary spastic paralysis, *IBMPFD* inclusion body myopathy with Paget’s disease and frontotemporal dementia, *LL* lower limb, *LMN* lower motor neuron, *N/A* not available, *PBP* progressive bulbar palsy, *PD* Parkinson’s disease, *PLS* primary lateral sclerosis, *PMA* progressive muscular atrophy, *POAG* primary open angle glaucoma, *PSP* progressive supranuclear palsy, *SCA2* spinocerebellar ataxia 2, *UL* upper limb, *UMN* upper motor neuron; *UN* unknown, *XD* X-linked dominant

## Genotype-phenotype correlations

### ALS1: SOD1

The first causative gene of ALS was identified as *SOD1* in 1993 [12]. Mutations in *SOD1* are very common, accounting for about 20 % of FALS and 1 ~ 2 % SALS cases [13]. To date, more than 185 disease-associated mutations have been described, spread throughout all the 5 exons of *SOD1* [14]. The majority of *SOD1* mutations are missense mutations, while small deletions or insertions are also described [15]. The pattern of inheritance is autosomal dominant except for the p.D90A mutation which is recessive in the Scandinavian population and dominant in others [16]. Among the *SOD1* mutations, p.D90A is the most common worldwide. However, regional disparity of *SOD1* mutations also exists. For example, the most frequent *SOD1* mutation in North America is p.A4V [17], but in the UK and Japan, the most common mutations are p.I113T, and p.H46R, respectively [18].

Overall, most patients with *SOD1* mutations develop a rapidly progressive ALS, although some cases show a diverse phenotype. AAO and severity may vary significantly depending on the variants involved. Patients with p.A4V, p.H43R, p.L84V, p.G85R, p.N86S, or p.G93A mutations exhibit an aggressive form of ALS with survival shorter than 3 years, while cases with p.G93C, p.D90A, or p.H46R mutations show longer life expectancies [18]. Cognitive impairment is very rare and bulbar onset is less frequent than in other FALS types [19]. Some cases with *SOD1* mutations have distinct clinical features. Patients carrying homozygous p.D90A mutation manifest insidious onset and a slow progression of ALS, with bladder involvement at the later stage [20]. In contrast, heterozygous p.D90A mutation is associated with various forms of ALS, including bulbar onset, upper-limb onset and fast progression, and lower-limb onset with fast progression [21, 22]. The p.A4V mutation causes a limb-onset aggressive form of ALS, with survival less than 2 years after the onset [23]. Cases with p.A4T mutation have a similar phenotype to that seen in p.A4V mutation [24]. We previously reported *SOD1* p.C111Y and p.G147D mutations in 3 Chinese ALS families. The p.C111Y mutation led to a relatively mild ALS phenotype, while the p.G147D was associated with a rapid progressive ALS [25]. The recently reported novel p.R115C mutation was identified in an ALS patient who had an extremely rapid progression and aggressive phenotype [14]. Another novel p.T137A mutation identified in two unrelated Italian families, however, causes a very slow progression of ALS [26, 27].

### ALS2: Alsin

Mutations in *ALS2* are responsible for autosomal recessive, early-onset forms of upper motor neuron diseases, such as infantile ascending hereditary spastic paraplegia (IAHSP) and juvenile primary lateral sclerosis (PLS) [28, 29]. To date, more than 50 patients with early onset (~1 year) of the disease have been reported to harbor *Alsin* mutations [18]. In the typical adult onset ALS, *Alsin* gene is rarely mutated [30]. Recently, a novel splice-site mutation (c.3512 + 1G > A) in *Alsin* was identified in a consanguineous JALS family with early onset anarthria and generalized dystonia [31].

### ALS4: senataxin (SETX)

ALS4 is a rare autosomal dominant form of juvenile-onset ALS due to mutations in *SETX* [32]. It is characterized by slowly evolving distal muscle weakness and atrophy, pyramidal signs, and sparing of bulbar and respiratory muscles [33]. In some cases, normal lifespan or atypical features are also described [34, 35]. A patient with *SETX* p.R2136C mutation presented with coexistence of ALS and inflammatory radiculoneuropathy [36]. We previously reported a missense mutation p.T1118I in a sporadic Chinese ALS patient who developed bulbar symptoms 3 years after the onset, and respiratory failure 2 years later [37]. In addition, recessive *SETX* mutations are reported to cause ataxia and oculomotor apraxia type 2 (AOA2) [38, 39].

### ALS5: spatacsin (SPG11)

*SPG11* mutations were recently identified in several juvenile-onset ALS cases, with autosomal recessive inheritance and AAO ranging from 7 to 23 years [18, 40]. Generally, the *SPG11*-associated ALS showed a slow progression and in some cases apparent UMN involvement [41]. In addition to ALS phenotype, mutations in *SPG11* also cause hereditary spastic paraplegia (HSP) with thin corpus callosum [42].

### ALS6: FUS

Mutations in *FUS* gene have emerged as the second most common cause of ALS, accounting for about 3 ~ 5 % FALS and ~1 % SALS [43, 44]. Up to now, more than 60 mutations in *FUS* have been identified in ALS cases (http://www.hgmd.org, accessed in March, 2015). Among these mutations, the majority are clustered in exon 15 which encode the C-terminus of the protein, and the most common one is p.R521C [15]. The inheritance is autosomal dominant aside from an autosomal recessive p.H517Q mutation and several *de novo* mutations [44].

The phenotypes associated with *FUS* mutations include adult-onset ALS, JALS, ALS-FTD, and rarely pure FTD [45]. Although most patients carrying *FUS* mutations exhibit a classical ALS phenotype without cognitive impairment, the clinical course of these ALS cases are diverse, even among carriers of the same mutations. Compared to *SOD1* patients, *FUS*-related ALS have an earlier AAO, more frequent bulbar disease, and a more rapid progression [46]. Some *FUS* mutations are also observed in patients with juvenile-onset ALS with AAO younger than 25 years [47, 48]. Atypical features such as ALS with mental retardation, ALS with parkinsonism and dementia [46], and essential tremor were seen in some patients with *FUS* mutations [49].

### ALS8: vesicle associated membrane protein B (VAPB)

*VAPB* mutation was firstly described in several Brazilian families with motor neuron degeneration of various patterns: late-onset spinal muscular atrophy, atypical ALS, or typical ALS [50]. Subsequently, five other point mutations and a small deletion were described. Overall, *VAPB* mutations are extremely rare in FALS. The phenotype-genotype correlation remains largely undetermined so far.

### ALS9: angiogenin (ANG)

The role of *ANG* mutations in ALS remains ambiguous. Approximate 30 *ANG* mutations have been reported in ALS, but only the p.K17II mutation is shown to co-segregate with the disease [51]. Mutations in *ANG* account for a small fraction of ALS cases. A few FALS cases are identified to have concomitant *ANG* mutations with *FUS* [52] or *SOD1* [53] mutations. In addition, some *ANG* mutations are detected in healthy controls [54]. A subset of *ANG* mutation carriers showed cognitive impairment suggestive of FTD [55], or Parkinson’s disease (PD) [56].

### ALS10: TARDBP

Mutations in *TARDBP* encoding TDP-43 account for 4 % of FALS cases and ~1 % of SALS [57, 58]. More than 50 mutations have been identified so far (http://www.hgmd.org, accessed in March, 2015), mostly clustered in the C-terminal region encoded by exon 6 of *TARDBP*. Mutations in *TARDBP* are predominantly missense with an autosomal dominant inheritance. Although *TARDBP* mutations are detected in ALS cases worldwide, some regional diversity does exist. For instance, the p.A382T mutation has been found in 28.7 % of all ALS cases in Sardinia [59].

Patients with *TARDBP* mutations usually exhibit a typical ALS phenotype, with limb or bulbar onset, variable disease course, and no overt dementia [60]. It is reported that the *TARDBP*–linked ALS has a trend for earlier AAO, more upper limb onset, and a longer duration, compared to SALS patients [52, 61]. However, we previously reported a *TARDBP* p.S292N mutation in a FALS case who developed dysarthria, dysphagia, and atrophy of lingual muscle at the age of 64 years [62]. The progression of ALS in this case seemed to be rapid. Other phenotypes associated with T*ARDBP* mutations include FTD [63], ALS-FTD [64], ALS with extrapyramidal signs [65], FTD with parkinsonism [66], and PD [67].

### ALS11: factor induced gene 4 (FIG4)

*FIG4* was previously implicated in Charcot-Marie-Tooth disease type 4J (CMT4J) [68]. Later, *FIG4* mutations were found in autosomal dominant FALS and SALS cases [69]. However, ALS was a rare phenotype of *FIG4* gene. The other phenotypes associated with *FIG4* mutations include PLS [69], Yunis–Varon syndrome [70], and familial epilepsy with polymicrogyria [71].

### ALS12: optineurin (OPTN)

Previously identified as the cause of primary open angle glaucoma (POAG) [72], mutations in *OPTN* have been found in both FALS and SALS cases in either a dominant or recessive manner [73]. Although *OPTN* mutations are relatively common in Japan ALS cases [73, 74], they are rare in Caucasian patients [75, 76]. The *OPTN*-related ALS showed relatively slow progression and long duration before respiratory dysfunction [73]. In addition to ALS phenotype, some patients with *OPTN* mutations present with extrapyramidal symptoms, aphasia, or FTD [77, 78].

### ALS13: Ataxin-2 (ATXN2)

Long (more than 33) CAG repeat expansion in *ATXN2* gene has been identified as a cause of spinocerebellar ataxia type 2 (SCA2) [79]. Recent studies demonstrated that intermediate expansion (27 ~ 33 repeats) of *ATXN2* was a significant risk factor for ALS [80–82]. However, whether the clinical features of ALS can be affected by *ATXN2* intermediate repeats is still controversial, because no correlation between *ATXN2* repeat length and AAO or survival was observed [83]. A few case reports have described motor neuron degeneration in SCA2 families, raising the possibility that motor neuron involvement is part of SCA2 [84, 85].

### ALS14: Valosin-containing protein (VCP)

The gene of *VCP* is known to be mutated in inclusion body myopathy with Paget disease of bone and frontotemporal dementia (IBMPFD) [86]. Recently, mutations in *VCP* were identified in patients with FALS or SALS [87]. Actually, *VCP* mutations are not a major cause of ALS. Although more than 38 mutations in *VCP* have been discovered (http://www.hgmd.org, accessed in March, 2015), only a few of them are responsible for ALS. The phenotype of patients with *VCP* mutations shows intra-familial variations from IBMPFD to FALS [88], or from ALS to FTD or ALS-FTD [87].

### ALS15: ubiquilin 2 (UBQLN2)

Mutations in *UBQLN2* were recently identified in X-linked dominant FALS [89]. However, *UBQLN2* mutations are not a frequent cause of ALS [90]. In the affected cases, incomplete penetrance has been noted in females [89]. The predominant phenotype associated with *UBQLN2* mutations is ALS, although several patients have concomitant symptoms of FTD [10]. The AAO has been reported to be significantly younger in males than in females, presumably because males are hemizygous but females are heterozygous for the mutation [89].

### ALS16: sigma non-opioid intracellular receptor 1 (SIGMAR1)

*SIGMAR1* mutations were recently identified in families affected with juvenile ALS [91] or ALS with dementia [92]. However, these findings have not been replicated by other groups, suggesting that *SIGMAR1* is a rare causative gene of ALS. Recently a splice-site mutation (c.151 + 1G > T) in *SIGMAR1* was reported to cause autosomal recessive distal hereditary motor neuropathy in a consanguineous Chinese family [93].

### ALS17: chromatin modifying protein 2B (CHMP2B)

Mutations in the *CHMP2B* gene were initially identified in patients with FTD [94] and then identified in patients with ALS [95, 96]. Since only several ALS cases with *CHMP2B* mutations were reported, there was no characteristic ALS clinical subtype associated with these patients. In addition, *CHMP2B* mutation (p.R69Q) was also identified in progressive muscular atrophy (PMA) [96].

### ALS18: profilin 1 (PFN1)

Missense mutations in *PFN1* are firstly reported in two large ALS families and 7 FALS patients [97]. Later, screenings of sizeable ALS and FTD cohorts from diverse populations demonstrated that *PFN1* mutations are a rare cause of ALS [98–100]. The reported cases with *PFN1* mutations seem to present classical ALS with limb onset and no evidence of FTD [97, 99, 101].

### ALS19: erb-b2 receptor tyrosine kinase 4 (ERBB4)

Recently, *ERBB4* was identified a causative gene of FALS19, which was characterized by typical, slowly progressive ALS and a lack of obvious cognitive dysfunction [102]. However, this finding has not been replicated by other groups. The genotype-phenotype correlations are thus not determined.

### ALS20: heterogeneous nuclear ribonucleoprotein A1 (hnRNPA1)

Mutations in *hnRNPA1* gene were recently identified in patients presenting with ALS and/or multisystem proteinopathy (MSP) [103]. However, subsequent studies failed to identify *hnRNPA1* mutations in patients with ALS, FTD, or MSP [103–105]. The associations of ALS with *hnRNPA1* mutations are still controversial.

### ALS21: matrin-3 (MATR3)

Several mutations in *MATR3* were recently reported to cause ALS [106]. Later, a heterozygous *MATR3* p.A72T mutation was identified in a sporadic ALS patient with bulbar onset [107]. In another study, 2 splicing variants and a missense mutation were identified in 3 ALS cases with AAO ranging from 58 to 79 years, and bulbar onset in 2 cases [108].

## ALS-FTD: C9ORF72

In 2011, two independent groups reported that the massive GGGGCC hexanucleotide repeat expansion in the non-coding regions of *C9ORF72* gene caused chromosome 9p-linked ALS and FTD [109, 110]. Currently, *C9ORF72* repeat expansions have become the most frequently genetic cause of FALS and familial FTD, accounting for about 40 and 25 % of the cases, respectively [13]. In families with ALS-FTD, the frequency reaches to 50–72 % [19]. Notwithstanding, the mutations seem to be geographically clustered, accounting for one third of FALS cases in Europe and North America, but a small percentage in Asian populations. Haplotype analysis indicates that a common European founder appears to be responsible for all cases [111].

The *C9ORF72* repeat expansions are associated with various phenotypes, including typical ALS, PMA, PLS, ALS-FTD, and pure FTD [44]. In patients with *C9ORF72* mutations, bulbar onset and cognitive impairment seems to be more frequent, and median survival is relatively lower than in patients carrying *TARDBP* or *SOD1* mutations [19]. However, there is no association between the repeat length and disease phenotype or AAO in *C9ORF72* mutation carriers [112]. In addition, the *C9ORF72* expansions also underlie a small portion of other neurological diseases, such as Alzheimer’s disease (AD) [113], Huntington’s disease (HD) [114], and PD [115].

## Other genes implicated in ALS

Several other genes are also implicated in ALS. *DCTN1* mutations were first identified in a family affected with LMN disease [116] and soon identified in several ALS families [117, 118]. Subsequently, a cluster of *DCTN1* mutations were identified in pedigrees with Perry syndrome [119], PD [120] or progressive supranuclear paralysis (PSP) [121], suggestive of phenotypic variability of *DCTN1* mutations. Mutations in *SQSTM1* were initially identified as a cause of Paget disease of bone (PDB) [122]. Recently, *SQSTM1* mutations were identified in ALS [123, 124] as well as FTD [125]. It is speculated that *SQSTM1* mutations are mainly associated with late-onset SALS, because the number of early-onset patients with *SQSTM1* mutations is much less than that of late-onset patients with *SQSTM1* mutations [126].

In addition, mutations in several other genes such as *DAO*, *UNC13A*, *NEFH*, *PRPH*, *TAF15*, and *ELP3*, have been reported as rare causes of ALS. For some genes, there are no additional reports about the mutations as a cause of ALS since the initial publications. Therefore, the evidence supporting a causative role of ALS is not fully convincing.

## Implication of the genotype-phenotype correlations

**Fig. 1:**
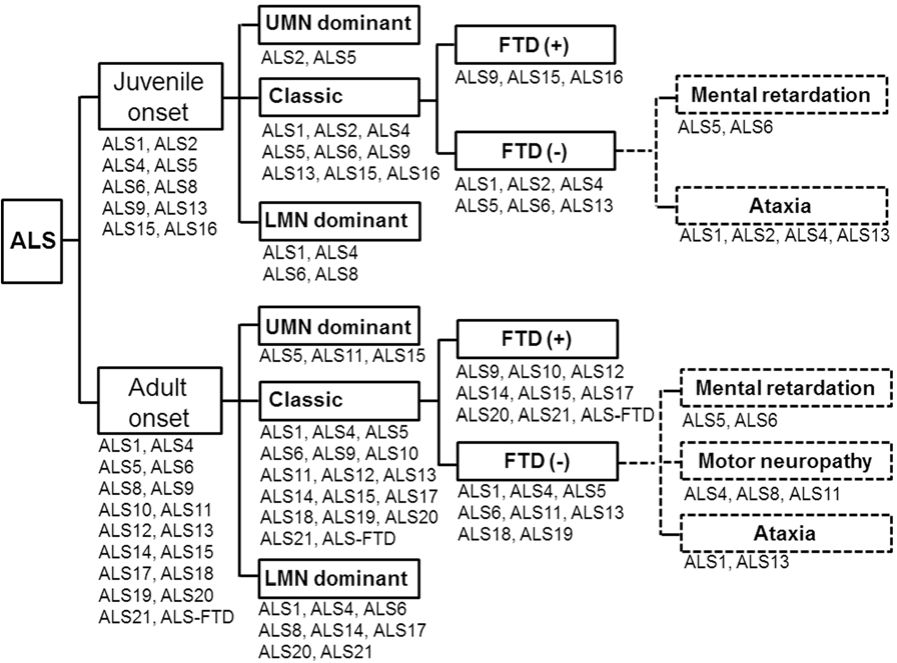
A flow diagram of genetic screening strategy in cases diagnosed with ALS

There is no a standard procedure to test the causative mutation in cases with ALS. Many factors should be considered, such as AAO, clinical features, progression, FTD involvement, inheritance manner, and even ethnicity. In this review, we summarized the possible genotype-phenotype correlations of ALS with the aim of providing some clue to improve the clinical decision. Here, we provided a flow diagram of genetic screening strategy in cases diagnosed with ALS (Fig. 1). AAO is an importance factor for the genetic investigations and can be divided into juvenile onset and adult onset. Juvenile onset is usually present in ALS1, ALS2, ALS4, ALS5, ALS6, ALS8, ALS9, ALS13, ALS15, and ALS16. For these cases with juvenile onset and UMN dominant symptoms, *Alsin*, *SPG11*, *SIGMAR1*, or *UBQLN2* might be a causative gene. In contrast, *FUS*, *VAPB*, *SOD1*, and *SETX* should be considered in cases with juvenile onset and LMN dominant phenotype. For these cases with juvenile onset and FTD symptoms, *ANG*, *UBQLN2*, and *SIGMAR1* can be investigated. In addition, *SPG11* and *FUS* can be sequenced in cases who also present mental retardation, while *SOD1*, *Alsin*, *SETX*, *ATXN2* can be considered in those cases with coexistence of cerebellar ataxia.

In cases with adult-onset ALS, many genes should be considered. Although the majority exhibit typical ALS features with both UMN and LMN symptoms, a fraction of cases present either UMN dominant symptoms or LMN dominant symptoms. *SPG11*, *FIG4*, *UBQLN2* may be mutated in cases with UMN dominant symptoms. In these cases with LMN dominant symptoms, mutations in *SOD1*, *SETX*, *FUS*, VA*P*B, *VCP*, *CHMP2B*, *hnRNPA1*, or *MATR3* might be identified. Presence of FTD hints possible mutations in *ANG*, *TARDBP*, *OPTN*, *VCP*, *UBQLN2*, *CHMP2B*, *hnRNPA1*, *MATR3*, or *C9ORF72*. Motor neuropathy involvement might occur in cases with mutations in *SETX*, *VAPB*, or *FIG4*. Complications of ataxia may be seen in cases with *SOD1* or *ATXN2* mutations.

Inheritance pattern also provides critical information as to the potential involvement of one or other specific genes in ALS. However, the diverse clinical features between intra-familial cases or a deceased parent at a young age may results in the appearance of “lack of familial history” in some cases. The presentation of ALS or FTD in first-degree relatives and the co-occurrence of ALS with FTD in some patients with ALS supported a positive family history. In addition, ethnic background should be considered when determining which genes are most likely. For example, *C9ORF72* has been regarded as the most common cause of ALS in Caucasians, but very rare in Asian population.

## Conclusions

Here, we outline the genotype-phenotype correlations of ALS. We hope this review is helpful for detecting the causative mutation in cases with ALS. Although a number of genes have been reported, many ALS cases still do not carry any mutation in the aforementioned genes. Other more genes might be identified as causative genes of ALS in the future.

## Abbreviations

AAO: age at onset
AD: Alzheimer’s disease
ALS: amyotrophic lateral sclerosis
ANG: angiogenin
AOA2: oculomotor apraxia type 2
ATXN2: ataxin-2
C9ORF72: chromosome 9 open reading frame 72
CMT4J: Charcot-Marie-Tooth disease
DCTN1: dynactin
ERBB4: erb-b2 receptor tyrosine kinase 4
FALS: familial amyotrophic lateral sclerosis
FIG4: factor induced gene 4
FTD: frontal temporal dementia
FUS: fused in sarcoma
HD: Huntington’s disease
hnRNPA1: heterogeneous nuclear ribonucleoprotein A1
HSP: hereditary spastic paraplegia
IAHSP: infantile-onset ascending hereditary spastic paralysis
IBMPFD: inclusion body myopathy with Paget disease of bone and frontotemporal dementia
JALS: juvenile amyotrophic lateral sclerosis
LMN: lower motor neuron
MATR3: matrin-3
MSP: multisystem proteinopathy
OPTN: optineurin
PD: Parkinson’s disease
PDB: Paget disease of bone
PFN1: profilin 1
PLS: juvenile primary lateral sclerosis
PMA: progressive muscular atrophy
PSP: progressive supranuclear paralysis
SALS: sporadic amyotrophic lateral sclerosis
SCA: spinocerebellar ataxia
SETX: senataxin
SIGMAR1: sigma non-opioid intracellular receptor 1
SOD1: superoxide dismutase 1
SPG11: spatacsin
SQSTM1: sequestosome 1
TARDBP: TAR DNA-binding protein
UBQLN2: ubiquilin 2
UMN: upper motor neuron
VAPB: vesicle associated membrane protein B
